# Evaluation eines frühen interdisziplinären multimodalen Assessments für Patienten mit Schmerzen: Protokoll einer randomisierten kontrollierten Studie (PAIN2020)

**DOI:** 10.1007/s00482-020-00497-3

**Published:** 2020-09-17

**Authors:** Ulrike Kaiser, Frank Petzke, Bernd Nagel, Ursula Marschall, Hans-Raimund Casser, Thomas Isenberg, Thomas Kohlmann, Gabriele Lindena, PAIN2020, Katharina Augustin, Carolin Althoff, Josef Heissenberger, Andreas Hölscher, Daniel Szczotkowski, Katja Schulz, Beatrice Metz-Oster, Jana Rensland, Lena Milch, Michael Pfingsten, Leonie Schouten, Karin Deppe, Anne Gärtner, Greta Hoffmann, Anke Preissler, Julia Pritzke-Michael

**Affiliations:** 1 Medizinische Fakultät und Universitäts SchmerzCentrum, Universitätsklinik Carl Gustav Carus Dresden, Fetscherstraße 74, 01307 Dresden, Deutschland; 2 Universitätsmedizin Göttingen, Schmerzmedizin, Klinik für Anästhesiologie, Universitätsmedizin Göttingen, Robert-Koch-Str. 40, 37075 Göttingen, Deutschland; 3 Ambulanz, Tagesklinik, Stationäre Behandlung, DRK Schmerz-Zentrum Mainz, Auf der Steig 16, 55131 Mainz, Deutschland; 4 Abteilung Medizin und Versorgungsforschung, BARMER, Lichtscheider Straße 89, 42285 Wuppertal, Deutschland; 5 Deutsche Schmerzgesellschaft e. V., Alt-Moabit 101b, 10559 Berlin, Deutschland; 6 Abteilung Methoden der Community Medicine, Institut für Community Medicine, Universitätsmedizin Greifswald, Walther-Rathenau-Str. 48, 17475 Greifswald, Deutschland; 7 PAIN2020, Berlin, Deutschland

**Keywords:** Schmerz und Risikofaktoren, Frühzeitige Diagnostik, Interdisziplinäre Multimodale Schmerztherapie, Prospektive klinische Studie, Versorgungsforschung, Pain and risk factors, Early interdisciplinary diagnostik, Interdisciplinary pain management, Prospective clinical study, Public health

## Abstract

Die Versorgung von Patienten mit Schmerzen und Chronifizierungsrisiko ist nach wie vor gekennzeichnet durch Über‑, Fehl- und Unterversorgung. Das Projekt PAIN2020 (Innovationsfonds 01NVF17049) hat zum Ziel, durch die Einführung eines frühzeitigen, auf Schmerz spezialisierten interdisziplinären diagnostischen Ansatzes die ambulante Versorgung von Patienten im Hinblick auf Schmerzen und das Funktionsniveau zu verbessern. Im Rahmen einer randomisierten kontrollierten Studie werden bundesweit in 31 Einrichtungen der Regelversorgung (schmerzspezialisiertes Angebot) Patienten mit Risikofaktoren bei bestehenden Schmerzen einer frühen Schmerzdiagnostik zugeführt. Die Interventionsbedingung besteht dabei in einem interdisziplinären multimodalen Assessment mit den beteiligten Disziplinen Schmerzmedizin, Physiotherapie und Psychologie. Die Kontrollbedingung umfasst einen einmaligen Termin bei einem Schmerztherapeuten der Qualitätssicherungsvereinbarung Schmerzmedizin bzw. mit Zusatzbezeichnung spezielle Schmerztherapie. Patienten und Vorbehandler erhalten entsprechend der Befunde detaillierte Empfehlungen für eine weitere bedarfsgerechte Behandlung. Es sind 2 Evaluationsansätze geplant. Für den ersten beträgt die zu erreichende Nettofallzahl 3840 Patienten, deren klinische Daten (Deutscher Schmerzfragebogen, zusätzliche Skalen) längsschnittlich erhoben (Einschluss, 3 und 6 Monate nach Diagnostik) und auf Grundlage eines Mehr-Ebenen-Modells ausgewertet werden. Im Rahmen eines 2. Ansatzes werden diese klinischen Daten einerseits um Sekundärdaten der BARMER ergänzt sowie die Patienten des ersten Evaluationsansatzes mit BARMER-Versicherten gematcht, die an dem Projekt nicht teilgenommen haben. Die Auswertung übernimmt ein externes Evaluationsinstitut. Das Projekt startete im April 2018.

## Abstract (en)

Health care provision for patients with pain and risk factors for chronicity is still insufficient and characterized in particular by over-, under- and misuse of existing approaches. The PAIN2020 project is funded by the German Federal Ministry of Health Care (*Innovationsfonds* 01NVF17049) and aims to improve health care delivery for this group of patients by implementing an early ambulatory diagnostic approach by a pain specialist, resulting in an improvement in pain and restored or maintained function. A randomized clinical trial in 31 facilities Germany-wide will identify eligible patients and guide them into early specialized pain diagnostics. The interventional arm provides an interdisciplinary multimodal assessment, delivered by pain medicine specialists, physiotherapists and clinical psychologists. The control arm contains a single assessment by a pain specialist. Patients and caregivers receive detailed recommendations for evidence-based interventions tailored to the needs of the individual patient. Two evaluation approaches will be merged. The first requires a net sample size of 3840 patients, assessed (admission) and followed-up (3 and 6 months) by clinical data (German Pain Questionnaire, additional scales) and analyzed by a multi-level approach. In a second evaluation arm the clinical data of the included patients will be supplemented by secondary data from a statutory health insurance (BARMER) and compared to a sample of policyholders not addressed by the study. Data analyses will be performed by an external evaluation institute. The project started in April 2018.

## Einleitung

Die Versorgung von Patienten mit Schmerzen wird immer wieder als unzureichend beschrieben [1]. Sie wird im Wesentlichen charakterisiert durch Überversorgung insbesondere hinsichtlich Medikamentengabe oder unimodaler, vor allem medizinischer Diagnostik und Therapie, Fehlversorgung durch fehlende Steuerung in bedarfsgerechte Therapieangebote bzw. durch mangelnde Anwendung bestehender Leitlinien, aber auch durch Unterversorgung im Hinblick auf aktivierende oder interdisziplinäre Angebote zu Beginn einer Schmerzerkrankung [2]. Insbesondere Risikofaktoren für eine spätere Chronifizierung von Schmerzen werden nach wie vor zu spät berücksichtigt. Dazu gehören u. a. persönliche Bewältigungsstile auf Seiten des Patienten (Krankheitskonzept, Durchhalten, Vermeidung von Aktivitäten), die häufig zu einer Fehlbewältigung (z. B. exzessives Schonen, Medikamentenfehlgebrauch, Überforderung) führen und damit die Zunahme sekundärer körperlicher und psychischer Prozesse (Disuse-Syndrom, Verstärkung von Kopfschmerzhäufigkeit, Depression etc.) begünstigen [3]. Weitere Risikofaktoren liegen in den derzeitig etablierten, primär unimodalen Strukturen der Versorgung von Patienten mit Schmerzen. Insbesondere die einseitige Diagnostik auf der somatischen Ebene vernachlässigt die multikausale Genese [4] und erzeugt selbst Folgen wie Hilflosigkeit, Rückzug und Aufgabe der eigenen Teilhabeanstrengungen.

Die diesbezüglich differenzierte Diagnostik von Risikopatienten zu einem frühen Zeitpunkt der Erkrankung wird bereits gefordert [2], kann aber aufgrund derzeitiger Strukturen der Versorgung nicht umgesetzt werden. Die frühzeitige Identifikation der Patienten vor einer Chronifizierung scheint entscheidend für den weiteren Krankheitsverlauf zu sein. Medizinische und psychosoziale Faktoren sollten gleichberechtigt berücksichtigt und eine sektorenübergreifende, bedarfsgerechte Steuerung der Patienten gewährleistet werden.

Bisherige Bestrebungen der Umsetzung im klinischen Alltag haben Limitationen für eine gute Versorgung verdeutlicht [5]. Die Beratung von Patienten mit Schmerzen zu Therapieangeboten erfordert entsprechend wissenschaftlicher Erkenntnisse der letzten Jahre einen anderen Umgang bereits zu einem Zeitpunkt, wenn sie erstmalig wegen Schmerzen einen Arzt aufsuchen. Eine Prüfung vorliegender Risikofaktoren sollte zu Beginn vorgenommen werden [2] und im entsprechenden Fall die diagnostische Berücksichtigung und Bewertung biopsychosozialer Faktoren am Schmerzgeschehen multiprofessionell und interdisziplinär erfolgen [2]. Diese Form der Diagnostik bedarf allerdings qualifizierter Strukturen und Voraussetzungen, die bisher in der ambulanten Versorgung nicht vorgehalten werden [6]. Die bedarfsgerechte Steuerung der betreffenden Patienten setzt das Vorhandensein von entsprechenden qualifizierten Therapieangeboten in erreichbarer Nähe der Patienten voraus, die entsprechend bestehender Empfehlungen für Rückenschmerz niederschwellig und ambulant interdisziplinär angeboten werden sollten [2]. Insgesamt sind die Voraussetzungen für die Versorgung von Patienten mit Schmerzen und Risiko einer Chronifizierung trotz der Empfehlungen von interdisziplinären Versorgungsformen [6–8] unter ambulanten Versorgungsbedingungen derzeit nicht ausreichend gegeben [1, 8, 9].

Dass solche Ansätze einen positiven Effekt auf verschiedene Aspekte des Erlebens der Patienten haben, konnte in Projekten zum Rückenschmerz nachgewiesen werden [10–14]. Kernelemente waren hier die frühzeitige Identifikation der Patienten sowie die Versorgung mit interdisziplinärer Diagnostik und anschließenden ambulanten interdisziplinären Versorgungsangeboten mit ausreichender Intensität. Dabei zeigte sich bereits, dass eine Arbeitsunfähigkeit von mehr als 60 Tagen eine eher schlechte Prognose für den weiteren Schmerzverlauf darstellte [15]. Patienten mit einer weniger fortgeschrittenen Chronifizierung benötigten demgegenüber weniger intensive Therapieangebote [11, 16], wobei allerdings bisher auf der bestehenden Datenbasis bei sehr unterschiedlichen Patientengruppen keine „Dosis-Wirkungs-Beziehung“ abzuleiten ist.

Das Projekt PAIN2020 (Patientenorientert.Abgestuft.Interdisziplinär.Netzwerk) soll die Effektivität eines frühen interdisziplinären multimodalen Assessments (IMA) für Patienten mit Schmerzen und Risiko einer Chronifizierung unter kontrollierten Bedingungen in der Versorgung prüfen. Ziel von PAIN2020 ist die Verbesserung der Versorgung dieser Patienten durch ein frühzeitiges schmerztherapeutisches Assessment und eine bedarfsgerechte Steuerung dieser Patienten in Angebote der Regelversorgung. Für die anschließende Versorgung der Patienten bieten die teilnehmenden Einrichtungen bei Bedarf in PAIN2020 ergänzend zur Regelversorgung 2 Formen niederschwelliger interdisziplinärer Therapiegruppen an. Die Identifikation entsprechender Patienten erfolgt sowohl mithilfe der beteiligten Krankenkasse (BARMER) als auch durch die Etablierung entsprechender Zuweisernetzwerke in der unmittelbaren Umgebung der teilnehmenden Einrichtungen. Für die Konzeption der Gruppentherapien werden Leitlinienempfehlungen (insbesondere in Bezug auf den nicht spezifischen Kreuzschmerz) und die Erfahrungen der bisher hauptsächlich teilstationär und stationär stattfindenden IMST berücksichtigt. Es erfolgt eine Anpassung von Inhalt und Umfang an das weniger chronifizierte Patientenkollektiv in PAIN2020.

## Methodik

### Projektstruktur

PAIN2020 ist ein Konsortialprojekt des Innovationsfonds (01NVF17049) mit einer Laufzeit von 36 Monaten.

*Konsortialführer* ist die Deutsche Schmerzgesellschaft e. V. Beteiligte *Konsortialpartner* sind die BARMER, ein externes Evaluationsinstitut (Institut für Community Medicine der Universitätsmedizin Greifswald), sowie 3 Einrichtungen mit Angeboten interdisziplinärer multimodaler Schmerztherapie (IMST; DRK Schmerz-Zentrum Mainz, Universitätsmedizin Göttingen, Universitätsklinikum Carl Gustav Carus Dresden). Das Konsortium verantwortet die Projektkonzeption, die Organisation mit Zentren- und Patientenrekrutierung, Unterstützung bei der lokalen Netzwerkbildung sowie das Monitoring der Studie.

Die Versorgung der Patienten im Interventionsarm der Studie erfolgt in PAIN2020-Zentren. PAIN2020-Zentren sind Einrichtungen, die bereits eine IMST anbieten bzw. die über die Voraussetzungen zu einer Zusammenarbeit der in der Schmerztherapie notwendigen Professionen nach den Empfehlungen der Deutschen Schmerzgesellschaft e. V. [6] verfügen. Die Ansprache von Versicherten und Patienten sowie deren Zuweisung in das Projekt wird sowohl durch Konsortialpartner (BARMER, Konsortium), durch die PAIN2020-Zentren selbst sowie durch kooperierende Ärzte (Hausärzte, Orthopäden etc.) durchgeführt.

### Beschreibung der neuen Versorgungsleistungen im Rahmen von PAIN2020

Die in PAIN2020 untersuchte interdisziplinäre multimodale Schmerztherapie [7] ist ein integrativer Versorgungsansatz aus somatischen, psychotherapeutischen und physiotherapeutischen Behandlungselementen, die in der Vorgehensweise aufeinander abgestimmt und auf einer gemeinsamen Philosophie begründet sind [17]. Das Team aus Ärzten, Psychologen und Physiotherapeuten arbeitet integrativ gleichberechtigt in regelmäßiger Abstimmung zu Diagnosen, Therapieplan und -verlauf. Die Anwendung evidenzbasierter Interventionen sowie eine standardisierte Dokumentation sind zusätzliche Qualitätsmerkmale dieser Versorgungsform. Das primäre Therapieziel besteht in der Wiederherstellung der subjektiven und objektiven Funktionsfähigkeit sowie in der Kontrollfähigkeit des Betroffenen [7].

Zur Struktur- und Prozessqualität als Grundvoraussetzung für die erfolgreiche Anwendung von IMST-Ansätzen [9] liegen zahlreiche Empfehlungen vor [7, 8, 17–20], die sich inzwischen auf verschiedene Sektoren der Versorgung beziehen [8]. Die neue Versorgungsform in PAIN2020 beinhaltet die Konzeption eines interdisziplinären multimodalen Assessments (IMA) unter Berücksichtigung der allgemeinen Kernmerkmale der IMST, erweitert um das Prinzip der Ergebnisoffenheit [6]. Das IMA als Steuerungstool dient der Steuerung der Patienten in bedarfsgerechte Versorgungsformen und ist als eigenständige Versorgungsform konzipiert [6, 21].

**Figure 1:**
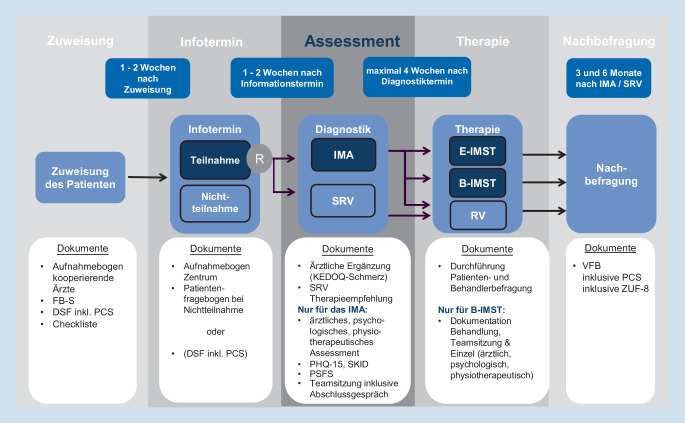

In PAIN2020 werden dementsprechend folgende neue Versorgungsleistungen eingeführt (Abb. 1):

- Screening der infrage kommenden Patienten auf Risikofaktoren durch kooperierende Ärzte, nach Zuweisung durch die BARMER oder nach Kontaktaufnahme durch die Patienten im PAIN2020-Zentrum.
- Eine frühzeitige, spezialisierte schmerztherapeutische Diagnostik: In einemIMA werden die Patienten hinsichtlich biopsychosozialer Faktoren zur Schmerzauslösung und -aufrechterhaltung ärztlich, physiotherapeutisch und psychologisch (einschließlich sozialer Faktoren) untersucht. Jede Profession nimmt ihre Befunde mit in eine integrierte Teamsitzung, wo sie gewichtet und Empfehlungen entsprechend der Erfordernisse der Beschwerdesymptomatik des Patienten formuliert werden. Im Anschluss an diese Teamsitzung besprechen alle beteiligten Professionen mit dem Patienten zusammen die erhobenen Befunde, Einschätzungen und Empfehlungen. Diese Vorgehensweise wurde aus den Empfehlungen aus [6] abgeleitet. Befund und Therapieempfehlungen werden an die ausführenden Behandler übermittelt. Die Dokumentation (einschließlich psychometrischer Befunde in der Vorbereitung zur Anamnese sowie Verlaufsbefragung zur Kontrolle) mit empfohlenen Instrumenten anerkannter Fachgesellschaften (z. B. Deutscher Schmerzfragebogen [22], MASK-P[23]) wird als Qualitätssicherung und als Teil der neuen Versorgungsleistung verstanden.
- Zwei ambulante Therapiemodule mit Edukation (E-IMST, einmaliger Termin in Gruppen zu 12 Patienten) oder Begleittherapie (B-IMST, 10 Termine in 10 Wochen in Gruppen zu 8 Patienten) bei entsprechendem im IMA festgestelltem Therapiebedarf. Beide Therapieformen sind bisher nicht Teil der Regelversorgung. Sie werden in PAIN2020 qualitativ und deskriptiv ausgewertet.

### Zielgruppe der Studie

In die Studie eingeschlossen werden BARMER-Versicherte ab einem Lebensalter von 18 Jahren, bei denen folgende *Einschlusskriterien* erfüllt sind:

Schmerzen unterschiedlicher Lokalisation, die trotz fachspezifischer Behandlung mindestens 6 Wochen lang bestehen oder die seit 2 Jahren (wenn auch in kürzeren Phasen als 6 Wochen) immer wieder auftreten, schmerzbedingte Einschränkungen im Lebensvollzug und der gesundheitsbezogenen Lebensqualität. Bei Berufstätigen kann eine schmerzbedingte Arbeitsunfähigkeit seit mind. 4 Wochen oder eine kumulierte Arbeitsunfähigkeit von mind. 6 Wochen in den vergangenen 12 Monaten als relevante schmerzbedingte Einschränkung gewertet werden. Diese Patienten weisen Risikofaktoren für die Chronifizierung von Schmerzen auf (siehe Tab. 1).

**Table 1:** 

*Biologische Risikofaktoren*	Bezüglich Lokalisation sich ausbreitende Schmerzen Hinweise auf Somatisierung (z. B. vielfältige, „bunte“ Symptomatik)
*Psychologisch-kognitiv-behaviorale Risikofaktoren*	Ausgeprägtes (verbales/nonverbales) Schmerzverhalten Schmerzfördernde Schmerzverarbeitung (Fokussierung, Ängste, …) Schmerzfördernde, krankheitsaufrechterhaltende Verhaltensweisen → ausgeprägtes Schon- und Vermeidungsverhalten → Überforderung, „Durchhalten“ → hohes Inanspruchnahmeverhalten im Versorgungssystem, Wunsch nach fortgesetzter Krankschreibung bzw. fortgesetzter Diagnostik
*Psychologisch-affektive Risikofaktoren*	Depressive Symptome im Erleben und/oder Verhalten Befindlichkeit geprägt durch Frustration/Ärger
*Soziale bzw. sozialmedizinische Risikofaktoren*	Hinweise auf Stressbelastung in Familie/Partnerschaft/sozialem Umfeld/Beruf Aktuelle Arbeitsunfähigkeit seit 4 Wochen bzw. kumulierte Arbeitsunfähigkeit von mindestens 6 Wochen in den vergangenen 12 Monaten

Der Einschluss Versicherter anderer Kassen ist darüber hinaus geplant.

Bei folgenden *Ausschlusskriterien* können Versicherte nicht an PAIN2020 teilnehmen: andere, akut wirksame schwerwiegende Erkrankungen, die z. B. eine aktivierende Behandlung verhindern, eindeutige „red flags“ mit Verdacht auf körperliche Risikofaktoren, manifeste chronische Schmerzerkrankung (Hinweise diesbezüglich sind: AU wg. Schmerzen länger als 6 Monate, schmerzrelevante Diagnose über mehr als 4 Quartale, vorhergehende Therapie mit starken Opioiden über eine Dauer von 3 Monaten, vorhergehende IMST in den letzten beiden Jahren), schwere und aktive psychiatrische Störung (z. B. Persönlichkeitsstörung, schwere Depression oder Angsterkrankung, Hinweise auf Suizidalität), Renten- oder Reha-Antrag, sprachliche und/oder kognitive Einschränkungen, die eine Durchführung des spezialisierten schmerztherapeutischen Assessments unmöglich erscheinen lassen.

Diese Kriterien werden während des Screenings sowie bei Prüfung der Einschlussfähigkeit im Zentrum erfragt und dokumentiert.

### Evaluation der Studie in PAIN2020

#### Design

PAIN2020 ist eine multizentrische randomisierte kontrollierte Studie mit 2 Studienarmen (Interventionsbedingung, Kontrollbedingung; siehe Abb. 1). Die Randomisierung in die primären Arme erfolgt stratifiziert nach Zentrum im Verhältnis 70 Interventionsbedingung (IMA und neue Versorgungsleistung): 30 Kontrollbedingung (schmerztherapeutische Regelversorgung, SRV). Die zentrumsbezogene blockweise Randomisierung erfolgt bei Studieneinschluss zentral und verblindet nach Übermittlung der pseudonymisierten Patientendaten in die PAIN2020-Datenbank. Die Studie ist als Längsschnittstudie mit 3 Messzeitpunkten konzipiert (Eingangserfassung, Nachbefragungen nach 3 und 6 Monaten).

**Figure 2:**
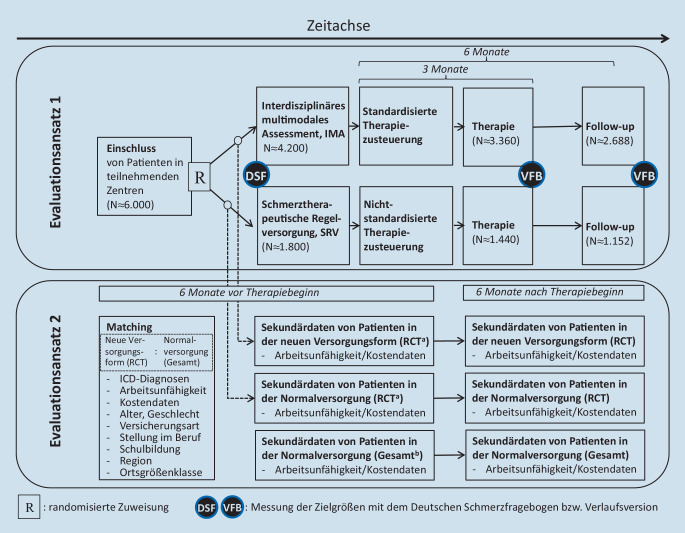

In Abb. 2 ist das Evaluationskonzept von PAIN2020 dargestellt. Es beinhaltet einen *Evaluationsansatz 1* (Vergleich klinischer Daten unter der Interventionsbedingung sowie der Kontrollbedingung) und einen *Evaluationsansatz 2* (Sekundärdaten des Kostenträgers für die randomisierten Versicherten und eine weitere Versichertengruppe).

##### Evaluationsansatz 1.

Für diesen Ansatz werden die Patienten nach schriftlicher Einwilligung randomisiert. Die Patienten der Interventionsbedingung erhalten das interdisziplinäre multimodale Assessment (IMA) und werden damit auf Grundlage der Teamentscheidung in die Regelversorgung bzw. die ergänzten Therapiearme E‑IMST und B‑IMST gesteuert. Die Patienten der Kontrollbedingung (schmerztherapeutische Regelversorgung, SRV) erhalten einen einmaligen schmerztherapeutischen Diagnostiktermin bei einem ärztlichen Schmerztherapeuten, der aufgrund seiner Befunde ebenfalls Empfehlungen für die weitere Behandlung im Rahmen üblicher Regelleistungen ausspricht.

##### Evaluationsansatz 2.

Hier werden die Sekundärdaten der BARMER für die Interventions- und Kontrollgruppe aus der randomisierten kontrollierten Studie (RCT) sowie einer weiteren Vergleichsgruppe in der Normalversorgung gegenübergestellt. Die Auswahl der weiteren Vergleichsgruppe aus dem Versichertenbestand der BARMER erfolgt anhand der in Abb. 2 aufgeführten Kriterien unter Verwendung des Propensity-score-matching-Verfahrens [24].

#### Zielgrößen

Für den *Evaluationsansatz 1* wurden als *primäre Zielgrößen* die *Veränderung der Schmerzintensität*, die *schmerzbedingte Funktionseinschränkung* sowie die *Einschätzung des Behandlungserfolgs* durch den Patienten definiert (siehe Tab. 2). Als Messzeitpunkte dienen der Einschluss der Patienten in die Studie (Ausgangswert) sowie 3 und 6 Monate nach dem Assessment. *Sekundäre Zielgrößen* sind die Reduktion des psychischen Disstresses und der Arbeitsunfähigkeitstage sowie die Veränderung in der gesundheitsbezogenen Lebensqualität.

**Table 2:** 

Variable			Messzeitpunkte			
Ziel	Variable	Datenquelle	Evaluationsansatz			
			2	1		
			Vor Projekteinschluss	T1 – Assessment	T2 – Therapieende bzw. 3 Monate	T3 – Follow up (6 Monate)
*Primäre Zielgrößen*	Schmerzintensität	Schweregradindex, von Korff (GCPS): Mittelwert aus 3 numerischen Ratingskalen (enthalten in DSF, VFB)	–	x	x	x
	Schmerzbedingte Funktionseinschränkung	Schweregradindex, von Korff (GCPS): Mittelwert aus 3 numerischen Ratingskalen (enthalten in DSF, VFB)	–	x	x	x
	Behandlungserfolg aus Sicht des Patienten	Globales Veränderungsitem (VFB Frage 13)	–	x	x	x
*Sekundäre Zielgrößen*	Psychischer Disstress	Depression-Angst-Stress-Skala (DASS) (enthalten in DSF, VFB)	–	x	x	x
	Arbeitsunfähigkeitstage	a) Bereitstellung durch BARMER	x	–	–	x
		b) Frage 11 in DSF Modul S und VFB Frage 11	x	x	x	x
	Gesundheitsbezogene Lebensqualität	VR-12 (enthalten in DSF und VFB Modul L)	–	x	x	x
	Katastrophisierung	Pain Catastrophizing Scale (PCS)	–	x	x	x
	Behandlungserfolg	Fragebogen zur Patientenzufriedenheit (ZUF 8)	–	–	x	x
	Frühberentung	Bereitstellung durch BARMER	x	–	–	x
	Direkte Kosten	Bereitstellung durch BARMER	x	–	–	x

*DSF* Deutscher Schmerzfragebogen (KEDOQ-Schmerz), *GCPS* Graded Chronic Pain Scale, *VFB* Verlaufsfragebogen (KEDOQ-Schmerz)

Für den *Evaluationsansatz 2* werden vergleichende Untersuchungen zwischen den beiden Gruppen von Studienteilnehmerinnen und -teilnehmern aus der randomisierten Studie sowie den Versicherten einer weiteren Vergleichsgruppe in Bezug auf zusätzliche *sekundäre Zielgrößen* vorgenommen. Hierzu zählen die bei der BARMER gemeldeten Arbeitsunfähigkeitszeiten, Frühberentungen sowie Daten zu direkten und indirekten Krankheitskosten (u. a. Arzneimittel, Heilmittel, stationäre Krankenhausbehandlung, Krankengeld).

#### Statistische Auswertung

Für die Fragestellung der Studie ist der Vergleich der beiden Studienarme in der randomisierten kontrollierten Studie im Hinblick auf die Veränderungen der 3 primären Zielgrößen im Zeitverlauf besonders relevant. Zur statistischen Analyse im *Evaluationsansatz 1* werden gemischte Modelle für wiederholte Messungen verwendet (u. a. Random-intercept-Modelle), in denen die Effekte der Faktoren Gruppenzugehörigkeit, Zeit und deren Wechselwirkung ggf. unter Berücksichtigung von Kovariaten modelliert werden.

Die Datenstruktur und das Grundmodell der statistischen Auswertung für den *Evaluationsansatz 2* entsprechen in wesentlichen Aspekten denen des Evaluationsansatzes 1. Durch die Betrachtung von nur 2 Zeitperioden (6 Monate vor bzw. nach Therapiebeginn) reduziert sich die Komplexität der Datenstruktur erheblich. Soweit besonders im Bereich der Kostendaten gravierende Abweichungen von der Normalverteilung bestehen, sollen diese durch geeignete Datentransformationen (z. B. Log-Transformation) verringert werden.

#### Fallzahlplanung

Die Berechnung der erforderlichen Stichprobengröße basiert auf den im *Evaluationsansatz 1* zur Untersuchung der 3 primären Zielgrößen geplanten Analysen. Ausgehend von einer Irrtumswahrscheinlichkeit für den Fehler 1. Art in einem 2‑seitigen Test mit einem Signifikanzniveau von *α* = 0,05 wird wegen der Korrektur für multiples Testen (3 Zielgrößen, 2 Messzeitpunkte) der *α*-Fehler nach Bonferroni auf *α* = 0,05/6 = 0,0083 adjustiert. Die Wahrscheinlichkeit, dass bei Vorliegen eines Gruppenunterschieds dieser auch statistisch erkannt wird (Power), soll 0,80 betragen. Wegen der komplexen Datenstruktur wurden zur Bestimmung der erforderlichen Stichprobengröße Simulationsrechnungen mit dem Program MLPowSim durchgeführt [25]. Es wurden mehrere Szenarios mit verschiedenen Annahmen über die Effektgrößen (10, 15 und 20 % der Residualvarianz) und über die Aufteilung der Varianzkomponenten auf die Ebene der Patienten und Zentren (100, 90 und 80 % auf der Ebene der Patienten) berechnet. Es zeigte sich, dass eine Nettostichprobengröße von insgesamt *N* = 3900 Fällen unter den gegebenen Annahmen ausreichend wäre, die Studienfragestellung zu beantworten. Nach Berücksichtigung zu erwartender Ausfallquoten von 20 % je Messzeitpunkt werden demnach initial insgesamt *N* = 6000 Fälle benötigt.

### Rekrutierung

Die Rekrutierung in PAIN2020 beinhaltet 3 Rekrutierungsebenen: 1. die PAIN2020-Zentrenrekrutierung, 2. die Patientenrekrutierung, 3. die Rekrutierung kooperierender Ärzte.

#### Zentrenrekrutierung

Die Studie soll bundesweit durchgeführt werden. 3 Zentren sind gleichzeitig Konsortial- und Kooperationspartner, sie definieren Inhalte und Ablauf und bieten die neuen Versorgungsleistungen selbst auch an. Nach einer breiten Information, einem einmaligen einführenden Workshop und dadurch erhaltenen verbindlichen Rückmeldungen werden die Qualifikationsvoraussetzungen für eine Einrichtung, an PAIN2020 teilzunehmen, im Kooperationsvertrag mit dem Konsortialführer festgelegt. Die Einrichtungen haben seit mindestens 3 Jahren Erfahrung in der Durchführung der interdisziplinären multimodalen Schmerztherapie entsprechend den Empfehlungen der Ad-hoc-Kommission Interdisziplinäre Multimodale Schmerztherapie der Deutschen Schmerzgesellschaft e. V. [7, 18]. Sie verfügen über strukturelle, personelle und prozessuale Voraussetzungen, die neue Versorgungsleistung umzusetzen. Ein Therapeutenteam (Primärdisziplinen bestehend in Medizin, Psychotherapie und Physiotherapie) mit im Organisationsplan dokumentierter Personalkonstanz führt regelmäßig integrative interdisziplinäre Teamsitzungen durch. Mindestens ein Vertreter je aus Medizin, Psychotherapie und möglichst auch der Physiotherapie verfügt an der Einrichtung über eine schmerztherapeutische Qualifikation nach einem Curriculum mit Anerkennung der schmerzbezogenen Fachgesellschaften der AWMF. In Ausnahmefällen, z. B. bei einem jungen Team oder Personalwechsel, können Abweichungen vereinbart werden.

Ein weiterer Vertrag wird mit der BARMER als Selektivvertrag zur Abrechnung der neuen Versorgungsleistungen nach SGB‑V §140a geschlossen.

Jedes rekrutierte Zentrum stellt auch die Zuweisung in die schmerztherapeutische Regelversorgung sicher; entweder durch schmerztherapeutische ärztliche Betreuung an der eigenen Einrichtung (wenn auch eine ambulante Ermächtigung und/oder eine Teilnahme an der Qualitätssicherungsvereinbarung Schmerztherapie vorliegt) oder mit einem externen Partner, der selber an der Qualitätssicherungsvereinbarung Schmerzmedizin teilnimmt.

#### Rekrutierung und Information kooperierender ambulanter Ärzte und Gesundheitsberufe

Einen wesentlichen Faktor in der frühzeitigen Versorgung der betreffenden Patienten stellt die rechtzeitige Identifikation von Risikofaktoren durch behandelnde Ärzte (u. a. Allgemeinmediziner, Orthopäden, Neurologen), Physiotherapeuten und andere Fachbereiche dar. PAIN2020-Zentren nehmen direkt Kontakt auf oder führen Informationsveranstaltungen durch. Diese Vernetzung ist im Sinne der Nachhaltigkeit der neuen Versorgungsleistung und damit wichtiger Bestandteil des Projektprotokolls.

#### Patientenrekrutierung

Es gibt die folgenden 3 Wege von Patienten in die Studie.

##### Über die BARMER und deren „Teledoktor“.

Die BARMER informiert Versicherte, bei denen die Versorgungsdaten den Beginn einer Chronifizierung nahelegen, postalisch über PAIN2020. Die Versicherten haben die Möglichkeit, beim Teledoktor (ein spezifisch geschulter telefonischer Beratungsdienst der BARMER) anzurufen, der erste Ein- und Ausschlusskriterien vorab prüft und den Anrufer dann an das PAIN2020-Zentrum direkt verbindet bzw. dem Patienten die Kontaktdaten des Zentrums zur Verfügung stellt. Durch das PAIN2020-Zentrum erhalten die Versicherten einen Termin für ein Informationsgespräch.

Zeitlich 1–2 Wochen vor Beginn der Rekrutierung von Versicherten im Bereich eines neuen Zentrums werden die Hausärzte der Region über das Projekt informiert. Auch sie erhalten die Möglichkeit, sich bei PAIN2020 über das Projekt und die Kooperationsmöglichkeiten mit ihrem Zentrum vor Ort detailliert zu informieren.

##### Über zuweisende ambulant tätige Ärzte (Niedergelassene und Krankenhausambulanzen) und Gesundheitsberufe.

Ärzte im Umkreis des PAIN2020-Zentrums werden eingeladen, Patienten entsprechend der Ein- und Ausschlusskriterien entweder direkt an ein PAIN2020-Zentrum zu schicken oder selbst zu screenen, über das Projekt zu informieren und an das betreffende Zentrum zu verweisen. Sie sind in erster Linie dem allgemeinärztlichen Fachbereich zuzuordnen, allerdings sind auch andere Fachbereiche oder andere Gesundheitsberufe (Physiotherapie, Pflege etc.) für die Patientenansprache von Relevanz.

##### Durch öffentliche Informationen über PAIN2020.

Die Information der Öffentlichkeit geschieht über die Homepage (www.pain2020.de) und verschiedene Medien. Bei Aktionen der BARMER werden interessierte Betroffene zunächst an den Teledoktor verwiesen, dort kurz informiert und an das regionale PAIN2020-Zentrum weitervermittelt. Bei Aktionen des PAIN2020-Zentrums können sich interessierte Betroffene auch direkt an das Zentrum wenden.

### Ablauf und Dokumentation

Die Dokumentation findet standardisiert ab dem ersten Patientenkontakt statt (siehe Abb. 1). Ab der Einwilligung werden die Daten patientenbezogen pseudonymisiert im Zentrum direkt vor Ort in die PAIN2020-Datenbank eingegeben. Eine Excel-Patientenliste (Excel, Microsoft Word; Microsoft, USA) fungiert als Masterdatei mit organisatorisch wichtigen Datumsangaben. Diese ist mit Benutzernamen und Kennwort SSL-verschlüsselt aufzurufen.

Im Folgenden werden die Stationen der Patienten beschrieben (systematische Kennung der Dokumentationsunterlagen, Abb. 1):

#### Screening beim behandelnden Arzt oder im PAIN2020-Zentrum (Abb. 1, Zuweisung)

Anhand eines Kurzfragebogens zur Einschätzung eines Chronifizierungsrisikos bei den Patienten sowie einer Checkliste werden die Risikofaktoren der Patienten (Tab. 1) erfasst. Bei Vorliegen von Risikofaktoren erhält der Patient einen Informationstermin im PAIN2020-Zentrum.

#### Informationstermin im PAIN2020-Zentrum (Abb. 1, Informationstermin)

Hier werden zunächst die Ein- und Ausschlusskriterien durch einen studienverantwortlichen Arzt geprüft und dann die Informationen zur Studie und zur besonderen Versorgung vermittelt. Die Patienten erhalten dazu einen Informationsbogen sowie eine Einwilligungserklärung sowohl für das Projekt PAIN2020 (als wissenschaftliche Studie mit Befragungen der Patienten zu Beginn und im Verlauf) als auch für die Einwilligung zur patientenbezogenen Abrechnung laut Selektivvertrag mit der BARMER (als Versorgungsleistung). Ein Arzt beantwortet aufkommende Fragen. Wenn Patienten ihre Einwilligung mit der Teilnahme schriftlich gegeben haben, werden sie zentral mithilfe der Excel-Patientenliste randomisiert und sie erhalten einen entsprechenden Assessmenttermin zum IMA oder zur SRV. Zur Vorbereitung des diagnostischen Termins (IMA bzw. SRV) erhalten die Patienten bereits den Deutschen Schmerzfragebogen [22] und die ergänzte Skala der Pain Catastrophizing Scale [26], die vor dem Assessment ausgewertet vorliegen sollen.

#### Schmerztherapeutisches Assessment (Abb. 1, Assessment)

Für das Assessment werden unabhängig von der Randomisierung als Grundlage für die Anamnese der Deutsche Schmerzfragebogen inklusive der enthaltenen psychometrischen Tests sowie der Pain Catastrophizing Scale ausgewertet sowie die ärztlichen Angaben zu Hauptschmerzlokalisation, Diagnosen, Chronifizierungsstadium und Vormedikation (in Wirkstoffgruppen) entsprechend dem KEDOQ-Schmerz-Kerndatensatz verwendet [27]. Auch die aus dem IMA resultierenden Empfehlungen an die Patienten und Behandler werden standardisiert dokumentiert und vom PAIN2020-Zentrum in die Datenbank eingegeben.

In der Interventionsgruppe mit IMA werden zusätzliche Fragebögen (Checkliste des strukturierten klinischen Interview für DSM-IV, SKID, [28]); Skala Somatisierung des Patient Health Questionnaire [29]) sowie die Befunde der 3 Berufsgruppen (einschließlich der multiaxialen Schmerzklassifikation für psychische Faktoren [23] sowie die patientenspezifische Funktionsskala [30]) und das Ergebnis der Teambesprechung mit dem Patienten (inklusive der Abweichung von den initialen Empfehlungen) dokumentiert.

#### Therapie im PAIN2020-Zentrum (Abb. 1, Therapie)

Die Therapie soll den Empfehlungen aus den Assessments folgen. Die Ausführung wird im Fall von Regelversorgung nicht gesondert dokumentiert, da diese über Krankenkassendaten im Nachgang nachvollzogen werden kann. Folgt dem IMA eines der neuen ambulanten Therapiemodule, wird deren zeitliche und gruppenbezogene Umsetzung dokumentiert. Im Fall der B‑IMST werden Einzeltermine und Teambesprechungen patientenbezogen dokumentiert.

#### Verlaufsbefragung (Abb. 1, Verlauf)

Alle Patienten werden vom aufnehmenden PAIN2020-Zentrum 3 und 6 Monate nach dem Assessment nachbefragt. Hierfür wurde der Verlaufsfragebogen um einen Zufriedenheitsfragebogen (ZUF 8, [31]) und die Pain Catastrophizing Scale (PCS, [26]) ergänzt. Als technische Hilfe für die Verlaufsbefragung gibt es neben dem Versand per Post die Option, den Patienten eine passwortgeschützte E‑Mail mit einem Link zuzuschicken.

### Datenmanagement und Datenschutz

Die medizinischen Befunde und persönlichen Informationen zu den teilnehmenden Patienten werden in der behandelnden Einrichtung für die Versorgung benötigt und als Versorgungsdaten 10 Jahre elektronisch gespeichert bzw. archiviert. Die Excel-Patientenliste (Masterdatei) beinhaltet darüber hinaus Daten, die für die Verlaufsübersicht vor Ort in den PAIN2020-Zentren notwendig sind, und wird daher ausschließlich in der entsprechenden Einrichtung gespeichert. Alle weiteren Daten werden pseudonymisiert in die PAIN2020-Datenbank eingegeben und dort gespeichert.

Der Einschluss von Patienten in der Studie erfolgt anhand dieser Excel-Patientenliste. Diese kommuniziert mit der PAIN2020-Datenbank über ein Pseudonym, das numerisch einem Hashwert aus Angaben in der Liste eindeutig den Patienten zugeordnet wird. Die Datenbank gibt eine eindeutige aufsteigende Patientenidentifikationsnummer zurück, die von der Einwilligung der Patienten an auf allen Dokumenten verwendet wird. Die patientenbezogene Eingabe erfolgt durch die PAIN2020-Zentren in dieser Datenbank. Die ausschließlich pseudonymisierten Datensätze bauen sich via Onlineformulare entsprechend dem Versorgungsverlauf der Patienten ab deren Einwilligung auf. Die Dokumente sind systematisch nummeriert und über ein Verzeichnis bei der Projektleitung abzurufen.

Die Patienten haben entsprechend der Datenschutzgrundverordnung (DSGVO) das Recht auf Rücknahme der Einwilligung, Auskunft, Berichtigung, Löschung oder Einschränkung der Verarbeitung.

PAIN2020-Zentren, die an KEDOQ-Schmerz teilnehmen, greifen für das IMA auf ihre Aufnahmeroutine im Rahmen von KEDOQ-Schmerz zu. Von KEDOQ-Schmerz wird der PAIN2020-Datenbank über den Hashwert der Patienten pseudonymisiert der Kerndatensatz übermittelt.

Die PAIN2020-Zentren haben Zugriffsrechte auf ihre eigenen Daten, das Projektteam auf die aller teilnehmenden Zentren in den Übersichten der PAIN2020-Datenbank, die Evaluatoren haben Zugang zu allen pseudonymisierten Projektdaten. Für die Auswertung von Daten sind Vereinbarungen im Kooperationsvertrag getroffen.

Für die Verwendung von Daten außerhalb PAIN2020 wird der KEDOQ-Ethikrat angerufen.

### Monitoring

Das Monitoring der Studie einschließlich der Therapiemodule erfordert einen erhöhten Aufwand, der darauf zurückgeht, dass 31 teilnehmende Zentren bundesweit einbezogen werden. Darüber hinaus ist PAIN2020 sowohl als klinische Studie als auch als Versorgungsangebot im Versorgungsalltag unter den Bedingungen eines Selektivvertrags zu verstehen. Damit sind Behandlungs- und Umsetzungsreinheit auf der einen Seite zur Sicherstellung interner und externer Validität der Ergebnisse ebenso zu berücksichtigen wie die Durchführbarkeit und Akzeptanz der Versorgungsleistung (Tab. 3). Dies ist eine Herausforderung unter den regionalen und hausinternen Bedingungen für den Ressourcenaufwand in jeder einzelnen Einrichtung sowie den Anforderungen einer komplexen Versorgungsleistung an sich.

**Table 3:** 

Variable			Bezugszeitpunkte			
Ziel	Variable	Datenquelle	Im Verlauf	T1 – Assessment	T2 – Therapieende bzw. 3 Monate	T3 – Follow up (6 Monate)
Prozess- und Strukturparameter	Erreichen der Zielpopulation Zuweisungsdokumentation	Screening (zus. Dokumentation)	–	x	–	–
	Randomisierung	Zus. Dokumentation Zentren	–	x	–	–
	Assessment biopsychosozialer Faktoren	Zus. Dokumentation Zentren	–	x	–	–
	Behandlungspfade	Soll-Ist-Vergleich (zus. Dokumentation Zentren)	–	x	x	–
	Durchführung der Versorgungsleistungen	Screening, Assessment, Empfehlungen und Therapiedokumentation (Zus. Dokumentation Zentren (Therapiemodule))	x	x	x	–
	Umsetzung der Qualitätsmerkmale	Vollständigkeit der Dokumentation Qualitätssicherung Struktur‑, Prozess- und Ergebnisparameter	x	x	x	x
	Durchführung der Gruppen	Teilnehmer, Dauer, Inhalte (zus. Dokumentation, Zentren)	x	–	–	–

Ziele des Monitorings sind zum einen die Sicherstellung der Umsetzungsreinheit des Projektprotokolls in den einzelnen teilnehmenden PAIN2020-Zentren unter Berücksichtigung unterschiedlicher Gegebenheiten und Voraussetzungen vor Ort. Darüber hinaus dient das Monitoring auch der Sicherstellung der Behandlungsreinheit als Voraussetzung der Stärke der Intervention einschließlich der damit hypothetisch angenommenen Effekte. Das dritte Ziel beinhaltet die Sammlung und Auswertung der Erfahrungen der Einrichtungen bei der Umsetzung der neuen Versorgungsleistung, die für die spätere mögliche Ausrollbarkeit von hoher Bedeutung sind.

Es sind derzeit die folgenden 4 Ebenen des Monitorings vorgesehen.

#### Persönliches Vor-Ort-Monitoring (Umsetzungsreinheit des Projektprotokolls)

Das erste von 3 persönlichen Monitorings findet im Rahmen der Sondierung in der Einrichtung statt. Ziel sind die Vermittlung von Information über PAIN2020 und die Begutachtung struktureller und prozessualer Aspekte (Qualifikationen, Räumlichkeiten, Personalsituation, Vertretungsregelungen, Vorbereitung bzw. Umsetzung der Abläufe für PAIN2020 etc.) zur Überprüfung der Einschlusskriterien für die Zentren zur Projektteilnahme.

Das zweite Monitoring findet nach Durchführung von mindestens 10 IMA statt. Ziele des zweiten Monitorings vor Ort sind in erster Linie administrative Überprüfungen der Einhaltung des Projektprotokolls (Dokumentationsqualität, korrekte und vollständige Durchführung der neuen Versorgungsleistung entsprechend Projektprotokoll, Einhaltung der Aufklärung und Archivierung der patientenbezogenen Dokumentationsunterlagen etc.). Den Zentren wird im Vorfeld ein ausführlicher Report zu den Inhalten des zweiten Monitorings zur Verfügung gestellt.

Das abschließende Monitoring wird als Abschlusskonferenz geplant, in der sowohl die Ergebnisse des Projekts direkt an die Zentren kommuniziert als auch die Erfahrungen der Teilnehmer am Projekt zusammengefasst und diskutiert werden.

#### Kontinuierliches Datenmonitoring (Umsetzungsreinheit, Behandlungsreinheit)

Die Dokumentation sowie die Durchführung der Studie werden über Kriterien aus den erhobenen Daten in der PAIN2020-Datenbank begleitet. Im Fall einer relevanten Abweichung vom Projektprotokoll wird mit dem betreffenden PAIN2020-Zentrum Kontakt aufgenommen und gemeinsam nach Lösungen gesucht. Im Vorfeld wurden sowohl Kriterien der Umsetzungsreinheit (hinsichtlich Projektprotokoll) als auch der Behandlungsreinheit (Durchführung der neuen Versorgungsleistungen IMA, E‑IMST und B‑IMST) erarbeitet und Indikatoren definiert, die Informationen zur Qualität der Umsetzung und Behandlung liefern und dem Projektteam anzeigen, wo vermehrter Unterstützungsbedarf besteht. Eine Zusammenfassung der wichtigsten Kriterien für die Umsetzungsreinheit und Behandlungsreinheit der neuen Versorgungsleistung sowie der Therapiemodule wird den Zentren in einem Turnus von 4 Monaten durch einen Report mitgeteilt. Bei Bedarf wird individuell Kontakt zu den Zentren aufgenommen, um Fragen oder Anpassungen am Vorgehen zu klären.

#### Fortlaufende Telefonkonferenzen mit den PAIN2020-Zentren

Für alle Themenfelder (Zuweisung, IMA, Dokumentation und Therapie) sind jeweils wiederkehrende (zwischen 4 und 6 Wochen), offene Telefonkonferenzen vorgesehen, in denen Fragen und Anliegen der PAIN2020-Zentren insbesondere zu Beginn der jeweiligen Projektphasen in den Zentren zeitnah und flexibel aufgegriffen werden. Gleichzeitig dient dieser Austausch der Vermittlung von Protokollanpassungen, generellen Rückmeldungen zum Projektverlauf von Seiten des Projektteams und der gemeinsamen Entwicklungen von Lösungen innerhalb der PAIN2020-Zentren. Diese Konferenzen werden protokolliert und die Protokolle werden allen Zentren in einem geschützten Onlinebereich der Projektwebseite zur Verfügung gestellt.

#### Telefonmonitoring nach 1) Projektbeginn (zu Dokumentation, Zuweisung, IMA, SRV) und nach 2) Therapie

Sowohl für die ersten Monate mit Zuweisung, IMA und SRV (einschließlich Dokumentation) als auch später im Projektverlauf für die Therapie werden einmalige, halbstandardisierte Telefoninterviews durchgeführt, um grundlegende Fragen der Umsetzung des Projektprotokolls und der Zufriedenheit der Zentren mit der Vorbereitung und Unterstützung vonseiten des Projektteams sowie grundlegende Hindernisse zu erfassen. Auch diese Informationen dienen einerseits der Beobachtung von Behandlungs- und Umsetzungsreinheit, aber auch der Möglichkeit, bei Schwierigkeiten zeitnah reagieren und das betreffende Zentrum bestmöglich und zeitnah individuell unterstützen zu können.

#### Übergeordneter Statusreport und individuelle Zentrumsreports

Im 4‑monatlichen Abstand wird ein übergeordneter Zentrumsreport zum Stand von PAIN2020 erstellt (Fallzahlaufkommen, Zuweisungswege, Patientenmerkmale, Durchführung IMA und Therapiemodule etc.). Dieser Report wird regelmäßig auf der PAIN2020-Homepage veröffentlicht und den PAIN2020-Zentren zugesendet.

Jedes PAIN2020-Zentrum erhält in einem Turnus von 4 Monaten zusätzlich einen individuellen Zentrumsreport, in der die Zusammenfassung der wichtigsten Parameter für das Gesamtprojekt im Allgemeinen und für jedes PAIN2020-Zentrum im Speziellen dargestellt wird. Anhand von Indikatoren zur inhaltlichen Validität und Plausibilität sowie Vollständigkeit wird bei relevanten Abweichungen mit dem PAIN2020-Zentrum nach geeigneten Lösungen gesucht. Der Zentrumsreport wird ausschließlich dem Projektteam und dem PAIN2020-Zentrum selbst zur Verfügung gestellt. Er wird nicht veröffentlicht.

### Ethik, Studienregistrierung und qualitative Betreuung der Projektdurchführung

Es liegt ein federführendes Ethikvotum der TU Dresden (EK216062018) sowie ein Votum zu einem Amendment vor. Entsprechend dieser Voten wurde für die Zentren entsprechend ihrer Anbindung (universitäre Einrichtungen bei den Ethikkommissionen der Universitäten, für Behandler der Normalversorgung bei den Ärzte- bzw. Landesärztekammern) Zweit- bzw. Übernahmevoten beantragt, die ausschließlich positiv befürwortet wurden. Zum Zeitpunkt der Manuskripteinreichung liegt für jedes Zentrum eine Ethikbestätigung vor.

Das Projekt ist im Deutschen Register für klinische Studien registriert (DRKS00015443).

Zur Beratung bezüglich der Studiendurchführung wurde ein Advisory Board eingesetzt, das sich aus Vertretern verschiedener Gremien der Deutschen Schmerzgesellschaft e. V. zusammensetzt.

### Aktueller Stand und Prognose für den Projektverlauf

#### Meilensteinplan für die Durchführung der Studie in PAIN2020

Das Projekt startete im April 2018. Für die Konzeption der Abläufe, Inhalte der Versorgungsleistungen, Dokumentation, Datenbank etc. war ein Zeitraum von 3 Monaten vorgesehen. Der erste Kooperationsvertrag und der erste Patient sollten im Juli 2018 (ein)geschlossen werden.

**Figure 3:**
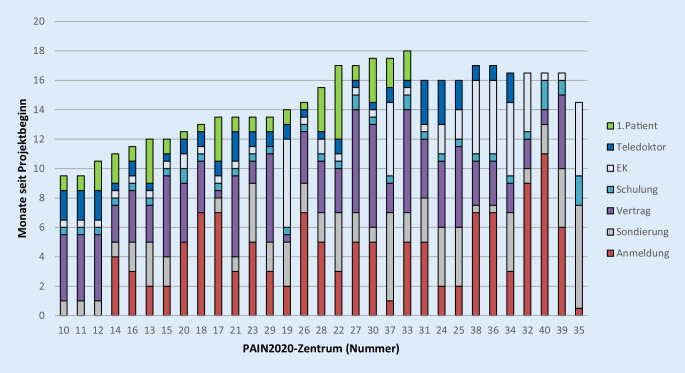

Die Konzeptionsphase verlängerte sich auf 9 Monate. Die Rekrutierungsphase der Zentren startete im Juli 2018 nach einem Workshop, die ersten Kooperationsverträge wurden ab Dezember 2018 unterzeichnet. Insgesamt, wie aus Abb. 3 ersichtlich, dauerte es aus unterschiedlichen Gründen zwischen 3 und 10 Monate, bis ein Zentrum PAIN2020 beitreten und die ersten Patienten einschließen konnte. Die Aufnahme von Einrichtungen in die Sondierungsphase wurde im Mai 2019 beendet, die laufenden Sondierungen wurden im September 2019 abgeschlossen.

#### Aktueller Stand zur Rekrutierung der PAIN2020-Zentren

**Figure 4:**
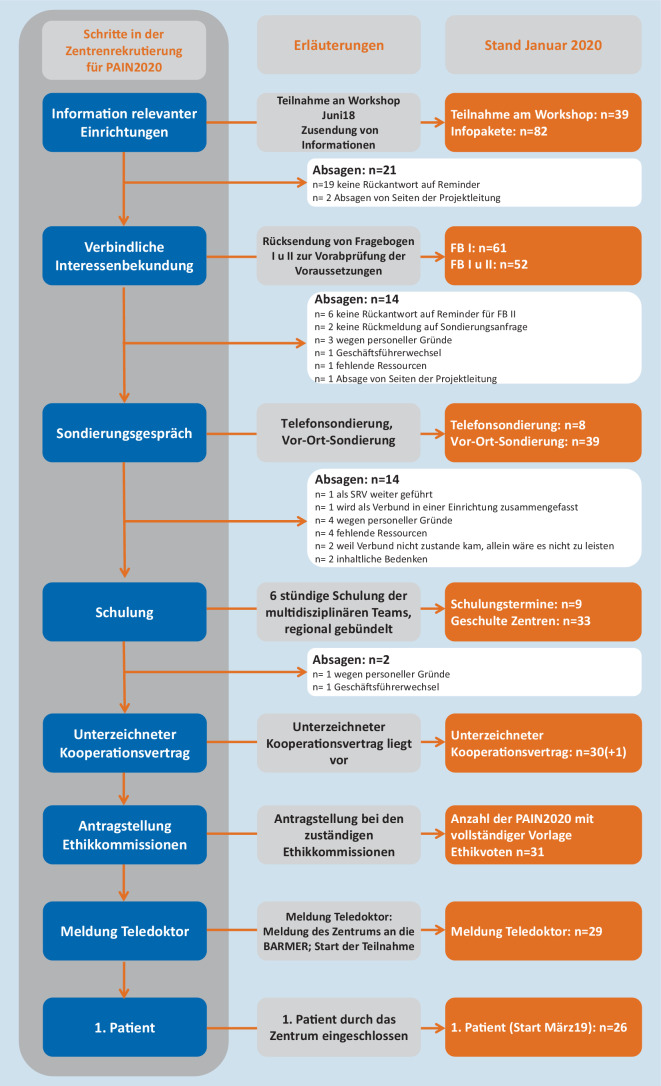

Zum Zeitpunkt der Manuskripteinreichung im November 2019 waren 31 Zentren in das Projekt aufgenommen (Abb. 4). Die PAIN2020-Zentren sind bundesweit verteilt (in 13 von 16 Bundesländern), 21 befinden sich in Großstädten, 4 in mittelgroßen Städten und 6 in ländlichen Regionen. 18 Einrichtungen verfügen über ein ambulantes sowie teilstationäres und/oder stationäres Angebot, 6 über ein stationäres oder teilstationäres Angebot, 7 arbeiten ausschließlich ambulant. Entsprechend der Klassifikationskriterien der Schmerzgesellschaften [32] waren nur 5 Zentren eindeutig zu klassifizieren. Gründe für eine nicht eindeutige Zuordnung waren die Qualifikation des nichtärztlichen Personals sowie die (fehlende) Teilnahme an Schmerzforschung.

Wie in Abb. 4 ersichtlich waren vom Zeitpunkt der verbindlichen Interessenbekundung eines Zentrums (Rücksendung der 2 Fragebögen zur Prüfung der Einschlusskriterien für teilnehmende Einrichtungen) 6 Schritte bis zur Aufnahme des ersten Patienten notwendig. Die aktuelle Fallzahl je Schritt (Stand November 2019) sowie die Gründe für das Ausscheiden von Interessenten sind dort ebenfalls aufgeführt. Von 82 angeschriebenen Einrichtungen bekundeten 52 ihr verbindliches Interesse. Es wurden 47 Einrichtungen sondiert und insgesamt 34 geschult. Von 31 liegt derzeit der unterschriebene Kooperationsvertrag vor. Es liegen für alle PAIN2020-Zentren Ethikvoten vor. Hauptsächliche Gründe für das Ausscheiden über den Rekrutierungszeitraum lagen in fehlenden Ressourcen und mangelnder Personaldecke für den zusätzlichen Aufwand in PAIN2020. Die erste Patientin wurde im März 2019 eingeschlossen.

## Ausblick

In diesem Artikel wird die Anlage der Studie einschließlich der vorbereitenden Arbeiten für PAIN2020 mit Stand November 2019 (Zeitpunkt der Ersteinreichung) geschildert. Damit soll ein in Leitlinien und Konzepten gefordertes interdisziplinäres multimodales Assessment gegenüber der Regelversorgung im randomisierten kontrollierten Vergleich geprüft werden. Die Hypothese geht von einer Überlegenheit einer interdisziplinär begründeten sektorenübergreifenden Empfehlung für Patienten mit Schmerzen und Risikofaktoren für deren Chronifizierung zur Verhinderung einer weiteren Chronifizierung aus.

Die bundesweite Anlage der Studie stellt eine große planerische und organisatorische Herausforderung dar. Auch die unterschiedlichen Settings der teilnehmenden PAIN2020-Zentren von Universitätskliniken bis hin zu (großen) Praxen stellt erhöhte Anforderungen an das Projektmanagement – nicht zuletzt durch die Anrufung der jeweils zuständigen Ethikkommissionen und der vielfältigen Szenarien zur Kombination der neuen Versorgungsform mit der Regelversorgung in den verschiedenen Settings. Gleichzeitig wird durch diese Vielfalt eine große Erfahrungsbreite gewährleistet, die eine spätere Umsetzung in die Regelversorgung möglicherweise erleichtert.

### Infobox 1 Einrichtungen, die an PAIN2020 teilnehmen

- GKH Bonn, Haus St. Petrus, Schmerztherapie
- Fachklinik Enzensberg, Interdisziplinäres Schmerzzentrum, Hopfen am See
- Brüderkrankenhaus St. Josef, Paderborn
- St. Vincenz Hospital Brakel, Klinik für Schmerzmedizin
- Universitätsklinikum Würzburg, Klinik und Poliklinik für Anästhesiologie
- Universitätsmedizin Göttingen, Georg-August-Universität, Abteilung Schmerzmedizin
- Universitätsklinikum Carl Gustav Carus an der Technischen Universität Dresden, UniversitätsSchmerzCentrum
- Universitätsklinikum Freiburg, Interdisziplinäres Schmerzzentrum, ISZ
- DRK-Schmerz-Zentrum Mainz
- REGIOMED-Klinikum Lichtenfels, Zentrum für Schmerzmedizin
- DRK-Kliniken Nordhessen Kassel, Klinik für Schmerzmedizin
- Universitätsklinikum Essen, Klinik für Neurologie
- Universitätsklinikum Jena, Sektion Schmerztherapie, Klinik für Anästhesiologie und Intensivmedizin
- Universitätsklinikum Hamburg-Eppendorf, Bereich Schmerzmedizin und Schmerzpsychologie
- Sankt Elisabeth KJF Klinik Neuburg/Donau
- DAS DOK:TOR Schriesheim
- AWO-Fachkrankenhaus Jerichow, Neurologie/Schmerztherapie
- Westmecklenburg-Klinikum Helene von Bülow Hagenow
- Reha-Zentrum Teltow
- Tabea Krankenhaus Hamburg, Schmerztherapie
- Asklepios Klinik Nord Hamburg, Zentrum für Interdisziplinäre Schmerztherapie
- Schmerzambulanz CVK, Charité, Klinik für Anästhesiologie m. S. Intensivmedizin Berlin
- Schmerzzentrum Rhein Main Frankfurt (Main)
- Westpfalz-Klinikum Kaiserslautern GmbH, Abteilung für Schmerztherapie
- St. Marienkrankenhaus Ludwigshafen, Schmerztherapie
- Zentralklinik Bad Berka GmbH, Zentrum für Interdisziplinäre Schmerztherapie
- Schmerz- und Palliativzentrum in Wiesbaden
- Praxis für ganzheitliche Schmerztherapie Hamburg
- Algesiologikum MVZ München
- AWO Fachkrankenhaus Jerichow
- Kreisklinik Jugenheim, Klinik für Orthopädie und Traumatologie, Sektion Konservative Orthopädie (ANOA), Manuelle Medizin u. Physikalische Therapie

## Fazit für die Praxis

Die bundesweite Anlage der Studie stellt eine große planerische und organisatorische Herausforderung dar. Sicher ist schon jetzt, dass alle Beteiligten – Projektteam und teilnehmende Zentren – mit viel Aufwand die Versorgungsforschung und die Versorgung für Patienten mit Schmerzen voranbringen.
